# Head to head comparison of the propensity score and the high-dimensional propensity score matching methods

**DOI:** 10.1186/s12874-016-0119-1

**Published:** 2016-02-19

**Authors:** Jason R. Guertin, Elham Rahme, Colin R. Dormuth, Jacques LeLorier

**Affiliations:** Department of Clinical Epidemiology and Biostatistics, McMaster University, Hamilton, ON Canada; Programs for Assessment of Technology in Health, St. Joseph’s Healthcare Hamilton, Hamilton, QC Canada; Research Institute of the McGill University Health Centre, Montreal, QC Canada; Department of Medicine, McGill University, Montreal, QC Canada; Department of Anesthesiology, Pharmacology & Therapeutics, University of British Columbia, Vancouver, BC Canada; Pharmacoeconomic and Pharmacoepidemiology unit, Research Center of the Centre hospitalier de l’Université de Montréal, Pavillon S, 850 St-Denis, 3e étage, Montreal, QC Canada

**Keywords:** Confounding by indication, Propensity scores, High-dimensional propensity scores

## Abstract

**Background:**

Comparative performance of the traditional propensity score (PS) and high-dimensional propensity score (hdPS) methods in the adjustment for confounding by indication remains unclear. We aimed to identify which method provided the best adjustment for confounding by indication within the context of the risk of diabetes among patients exposed to moderate versus high potency statins.

**Method:**

A cohort of diabetes-free incident statins users was identified from the Quebec’s publicly funded medico-administrative database (*Full Cohort)*. We created two matched sub-cohorts by matching one patient initiated on a lower potency to one patient initiated on a high potency either on patients’ PS or hdPS. Both methods’ performance were compared by means of the absolute standardized differences (ASDD) regarding relevant characteristics and by means of the obtained measures of association.

**Results:**

Eight out of the 18 examined characteristics were shown to be unbalanced within the *Full Cohort*. Although matching on either method achieved balance within all examined characteristic, matching on patients’ hdPS created the most balanced sub-cohort. Measures of associations and confidence intervals obtained within the two matched sub-cohorts overlapped.

**Conclusion:**

Although ASDD suggest better matching with hdPS than with PS, measures of association were almost identical when adjusted for either method. Use of the hdPS method in adjusting for confounding by indication within future studies should be recommended due to its ability to identify confounding variables which may be unknown to the investigators.

**Electronic supplementary material:**

The online version of this article (doi:10.1186/s12874-016-0119-1) contains supplementary material, which is available to authorized users.

## Background

Observational studies provide real world information on drug use and their potential effect on health outcomes but are prone to confounding by indication [1–4]. The traditional propensity score (PS) method is often used to control for confounding by indication. It represents “the conditional probability of assignment to a particular treatment given a vector of observed covariates” and is generally obtained thanks to a logistic regression model [5].

The high-dimensional propensity score (hdPS) method has recently been proposed and has been rapidly and widely adopted to address confounding by indication [6, 7]. Unlike the PS method which is limited to investigator-specified covariates; the hdPS method also uses a computerized algorithm to select a large number of potential confounders contained within the examined database [5, 7].

It is of interest to compare the performance of these two methods in controlling for confounding by indication to inform the design of future observational studies. Performance of both methods may be compared using two distinct approaches, 1) by examining the balance achieved on key potential confounders between sub-cohorts matched on these two scores [4, 8–11], and 2) by comparing the measures of associations obtained from the matched sub-cohorts to a gold standard comparator [7, 12–14].

Recently, results of a meta-analysis of randomized controlled trials (RCT) have found that exposure to higher statin doses might be associated with higher risks of diabetes (Odds ratio [OR] =1.12 [95 % confidence intervals (CI) 1.04–1.22]) [15]. Although results obtained from observational studies have been conflicting [16], four out of five published studies found a small increased but statistically significant dose-dependent relationship [17–20]. However, it is possible that in those observational studies, patients at higher risk of diabetes were more likely to be initiated on higher statins doses: a classic example of confounding by indication offering an excellent opportunity to compare the relative performance of these two scores. In this study, we aim to compare the performance of the PS and hdPS methods in adjusting for confounding by indication using the two approaches defined above.

## Methods

### Data sources

This study was performed using medico-administrative databases from the province of Quebec, Canada. Quebec is the second most populated province in Canada, with more than 8 million inhabitants [21]. A unique identification number is assigned to every individual, and all diagnoses and all health services provided are systematically recorded within the *Régie de l’assurance maladie du Québec* (RAMQ) databases. Pharmaceutical claims are also recorded but only for residents covered by the RAMQ public drug insurance plan. Information was obtained from the Quebec physician’s service and claims databases (i.e. RAMQ databases) and the Quebec hospitalisation databases (i.e., *Maintenance et Exploitation des Données pour l’Étude de la Clientèle Hospitalière* [MED-ECHO] databases), which have previously been validated [22–25]. For this study we used three RAMQ databases (i.e., the Demographic, Medical Services and Claims and Pharmaceutical databases) and three MED-ECHO databases (i.e. the Hospitalisation Descriptions, Diagnoses and Intervention databases). Patient records were linked across all databases by use of the unique identification number. The identification numbers were encrypted to protect patient confidentiality. Access to data was granted by the *Commission d’accès à l’information* and the protocol was approved by the *Centre hospitalier de l’Université de Montréal* ethics’ committee.

### Full Cohort

RAMQ provided us with a cohort of 800,551 incident statin users; the date of the first statin dispensation was defined as the cohort entry date. Patients were considered to be incident statin users if they did not have a claim for a statin dispensation in the year prior to the cohort entry date. Eligible patients had: 1) to have been newly initiated on either simvastatin, lovastatin, pravastatin, fluvastatin, atorvastatin or rosuvastatin between January 1^st^ 1998 and December 31^st^ 2010, 2) to be covered by the RAMQ public drug insurance plan for at least a year prior to the cohort entry date and 3) to be at least 40 years of age at the cohort entry date. We excluded every patient who, in the year prior or on the cohort entry date: 1) received any other cholesterol lowering drug dispensation (including niacin, cerivastatin or a combination statin drug); 2) received a dispensation for drugs used in the treatment of diabetes (WHO ATC A10) [26]; 3) received a diagnosis of diabetes (ICD-9 code: 250.x; ICD-10 codes: E10.x – E14.x); 4) were admitted in a long-term care facility or 5) received >1 statin dispensation on the cohort entry date. Patients who met both inclusion and exclusion criteria were entered within the *Full Cohort*.

### Exposure status

Patients were categorized into two categories based on the dose and relative potency of the first statin dispensation they received [18, 27]. Patients whose first statin dispensation was for a daily dose of ≥10 mg of rosuvastatin, ≥20 mg of atorvastatin or ≥40 mg of simvastatin formed the high potency group and remaining patients formed the lower potency group.

### Outcome status

Every patient who received either a dispensation of a drug used in the treatment of diabetes (WHO ATC A10) or a diagnosis of diabetes (ICD-9 code: 250.x; ICD-10 codes: E10.x – E14.x) within the 2 years following the cohort entry date was defined as a case, all other patients were considered to be diabetes-free.

### Propensity score method

We used the same list of variables that were used by Dormuth and colleagues to create the PS model [18]. This list included the following covariates: patients’ sex, age and poverty level status (yes versus no) at the cohort entry date, year of entry within the cohort (as a categorical variable), medical resource utilization variables (≥1 hospitalisation, ≥5 outpatient visits, ≥5 distinct drugs dispensed to the patient, all within the year prior to the cohort entry date), drug dispensation variables (dispensation of loop diuretics, dispensation of acetaminophen, dispensation of calcium blockers, dispensation of beta-blockers, dispensation of angiotensin receptor blockers and dispensation of angiotensin converting enzyme inhibitors, all in the year prior to the cohort entry date) and comorbidity variables (hypertension, hypercholesterolemia, myocardial infarction (MI), stroke, peripheral vascular disease (PVD), congestive heart failure, coronary artery bypass graft, and percutaneous coronary intervention (PCI), all in the year prior to the cohort entry date).

Following the selection of the PS model, patients’ PS were assessed for all patients included within the *Full Cohort*. Trimming was performed and patients located within non-overlapping regions of the PS distribution were excluded from the analysis, all other patients were eligible for inclusion within the *Matched PS Sub-Cohort* [28]. Lower potency controls were found for patients in the high potency group using a greedy, nearest neighbor 1:1 matching algorithm. Matching occurred if the difference in the logit of PS between nearest neighbors was within a caliper width equal to 0.2 times the standard deviation (SD) of the logit of the PS [29]. Patients selected by the matching algorithm were included within the *Matched PS Sub-Cohort*.

### High-dimensional propensity score method

hdPS were estimated for all patients included in the *Full Cohort* [7]. Detailed description of the hdPS method can be found elsewhere [7]. Briefly, the construction of the hdPS model involves two processes, 1) investigators select covariates to be forced within the hdPS model (similar to what is done within an investigator-specified PS model) and 2) the hdPS algorithm selects an additional list of covariates measured within the selected data dimensions based on their multiplicative bias assessment which is then also included within the final hdPS model. Within this study, estimation of patients’ hdPS were conducted using the default setting of the SAS hdPS macro v.1 (i.e., top 200 most prevalent variables per data dimensions, top 500 binary empirical covariates based on multiplicative bias assessment).

We structured the data collected from the year prior to the cohort entry date from the following 6 data dimensions: 1) drugs dispensed in an outpatient setting, 2) physician claims codes for inpatient and outpatient procedures, 3) physician claims for inpatient and outpatient diagnostic codes, 4) specialty of the physician providing care, 5) hospitalisation discharge data for inpatient procedure codes and 6) hospitalisation discharge data for inpatient diagnostic code.

In addition to the 500 variables selected by the default option of the hdPS algorithm [7], we forced the following covariates within the hdPS model: patients’ sex, age and poverty level status (yes versus no) at the cohort entry date, year of entry within the cohort (as a categorical variable), medical resource utilization variables (≥1 hospitalisation, ≥5 outpatient visits, ≥5 distinct drugs dispensed to the patient, all within the year prior to the cohort entry date). These variables were forced in the model since they could not be selected by the SAS hdPS algorithm v.1 we were using. Trimming was performed and patients located within non-overlapping regions of the hdPS distribution were excluded from the analysis [28], all other patients were eligible for inclusion within the *Matched hdPS Sub-Cohort*. Lower potency controls were found for patients in the high potency group using a greedy, nearest neighbor 1:1 matching algorithm. Matching occurred if the difference in the logit of hdPS between nearest neighbors was within a caliper width equal to 0.2 times the SD of the logit of the hdPS [29]. Patients selected by the matching algorithm were included within the *Matched hdPS Sub-Cohort.*

### Statistical analyses

Absolute standardized differences (ASDD), defined as the absolute between group difference over the pooled SD of the two groups, were used to compare patient characteristics between patients exposed to a high potency versus lower potency statin within the *Full Cohort* and both sub-cohorts [4, 8–11]. ASDD < 0.1 are generally assumed to indicate good balance between groups [2, 10]. Discrete data are presented in absolute and relative values (n [%]) and continuous data are presented as mean (SD). OR (95%CI) of diabetes occurrence in the high over lower potency statin groups were estimated within the *Full Cohort* and within both matched sub-cohorts; no adjustment beyond matching was performed.

All statistical analyses were conducted with SAS version 9.3 (SAS Institute, Cary, North Carolina).

## Results

### Characteristics of the patients included within the Full Cohort

**Fig. 1:**
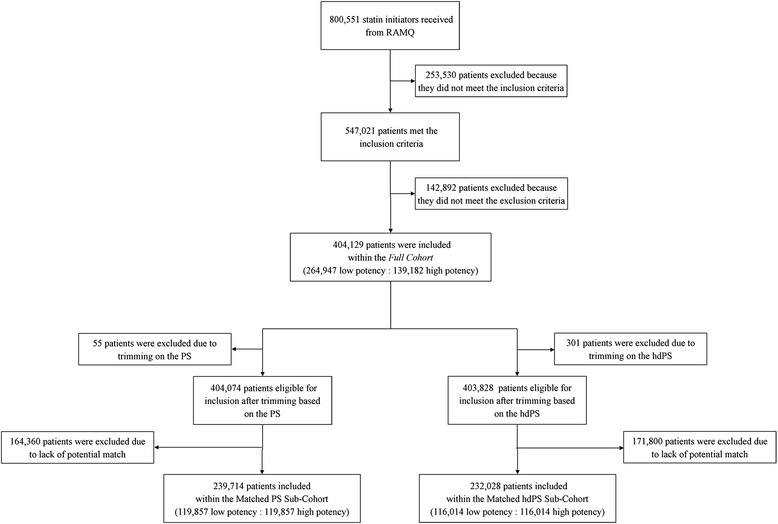
Patient flow-chart within the study. hdPS, High-dimensional propensity score; PS, Propensity score

Figure 1 shows the flow chart of patients included within the *Full Cohort*, the *Matched PS Sub-Cohort* and the *Matched hdPS Sub-Cohort*.

Baseline characteristics of the *Full Cohort* are shown in Table 1. Among the 404,129 patients included within the *Full Cohort*, 264,947 patients (65.6 %) were dispensed a lower potency statin and 139,182 patients (34.4 %) were dispensed a high potency statin on the cohort entry date. About half of patients (192,964 [47.8 %]) were males and the average age was 65.2 years (SD 11.0). Among the 18 examined patient characteristics, eight (44.4 %) were shown to have an ASDD > 0.1 indicating the presence of unbalance. History of a PCI (ASDD = 0.30) and history of a MI (ASDD = 0.27), both in the year prior to the cohort entry date, showed the greatest degree of imbalance (Table 1). In addition, onset of diabetes within 2-years follow-up was identified in 12,978 patients (3.2 %) of the 404,129 patients included within the *Full Cohort*.

**Table 1:** Demographic characteristics and comorbidity status of the *Full Cohort* at baseline

	Low potency n (%)	High potency n (%)	Absolute standardized differences
	264,947 (100.0)	139,182 (100.0)	
Age, mean (SD)^a^	65.6 (10.9)	64.5 (11.3)	0.098
Male sex	118,262 (44.6)	74,702 (53.7)	0.181
At least 5 medical outpatient visits	170,234 (64.3)	77,032 (55.4)	0.182
At least 1 hospitalisation	59,591 (22.5)	45,777 (32.9)	0.234
Myocardial infarction	15,056 (5.7)	18,899 (13.6)	0.270
Stroke	7150 (2.7)	5480 (3.9)	0.069
Hypertension	110,508 (41.7)	59,705 (42.9)	0.024
Hypercholesterolemia	88,458 (33.4)	47,005 (33.8)	0.008
Peripheral vascular disease	5446 (2.1)	3338 (2.4)	0.023
Congestive heart failure	11,337 (4.3)	8830 (6.3)	0.092
Coronary artery bypass graft	3589 (1.4)	3189 (2.3)	0.070
Percutaneous coronary intervention	7742 (2.9)	14,089 (10.1)	0.295
Dispensation of loop diuretics	16,612 (6.3)	10,188 (7.3)	0.042
Dispensation of calcium blockers	64,569 (24.4)	32,192 (23.1)	0.029
Dispensation of beta-blockers	77,669 (29.3)	49,147 (35.3)	0.128
Dispensation of angiotensin receptor blockers	35,741 (13.5)	25,325 (18.2)	0.129
Dispensation of angiotensin converting enzyme inhibitors	52,563 (19.8)	36,030 (25.9)	0.144
At least 5 different drugs dispensed	151,395 (57.1)	84,503 (60.7)	0.073

Comorbidity status, drug dispensations and medical utilization rates were assessed in the year prior to the cohort entry date. Absolute standardized differences are defined as the between group difference over the pooled standard deviation of the two groups

^a^At the cohort entry date

### Characteristics of patients included within the Matched PS Sub-Cohort

PS were calculated for all 404,129 patients included within the *Full Cohort* (kernel density PS curves for all patients included within the *Full Cohort* are provided in Additional file 1). Fifty-five (0.0 %) patients, 33 (0.0 %) lower potency and 22 (0.0 %) high potency, had PS located within non-overlapping regions and were excluded from the analysis. Among the remaining 404,074 patients, we matched 119,857 patients (29.7 %) initiated on a high potency statin to 119,857 patients (29.7 %) initiated on a lower potency statin based on their individual PS; selected patients formed the *Matched PS Sub-Cohort* (Fig. 1). This sub-cohort was comprised of 119,931 male patients (50.0 %) and the average age was 64.7 years (SD 11.2) (Table 2). Balance was obtained for all 18 examined patient characteristics (ASDD ranged from 0.002 to 0.031 with an average of 0.015).

**Table 2:** Demographic characteristics and comorbidity status of the *Matched PS Sub-Cohort* at baseline

	Low potency n (%)	High potency n (%)	Absolute standardized differences
	119,857 (100)	119,857 (100)	
Age, mean (SD)^a^	64.6 (11.2)	64.8 (11.2)	0.021
Male sex	59,690 (49.8)	60,241 (50.3)	0.009
At least 5 medical outpatient visits	68,696 (57.3)	69,017 (57.6)	0.005
At least 1 hospitalisation	29,527 (24.6)	31,129 (26.0)	0.031
Myocardial infarction	8457 (7.1)	8527 (7.1)	0.002
Stroke	3824 (3.2)	4219 (3.5)	0.018
Hypertension	49,335 (41.2)	50,719 (42.3)	0.023
Hypercholesterolemia	38,760 (32.3)	38,887 (32.4)	0.002
Peripheral vascular disease	2374 (2.0)	2691 (2.3)	0.018
Congestive heart failure	5412 (4.5)	5852 (4.9)	0.017
Coronary artery bypass graft	1756 (1.5)	1988 (1.7)	0.016
Percutaneous coronary intervention	5255 (4.4)	4805 (4.0)	0.019
Dispensation of loop diuretics	7202 (6.0)	7775 (6.5)	0.020
Dispensation of calcium blockers	26,878 (22.4)	27,928 (23.3)	0.021
Dispensation of beta-blockers	35,805 (29.9)	36,741 (30.7)	0.017
Dispensation of angiotensin receptor blockers	21,228 (17.7)	21,776 (18.2)	0.012
Dispensation of angiotensin converting enzyme inhibitors	25,537 (21.3)	26,484 (22.1)	0.019
At least 5 different drugs dispensed	69,608 (58.1)	70,087 (58.5)	0.008

Comorbidity status, drug dispensations and medical utilization rates were assessed in the year prior to the cohort entry date. Absolute standardized differences are defined as the between group difference over the pooled standard deviation of the two groups

^a^At the cohort entry date

### Characteristics of patients included within the Matched hdPS Sub-Cohort

Three hundred and one (0.1 %) patients, 54 (0.0 %) lower potency and 247 (0.1 %) high potency, had hdPS located within non-overlapping regions and were excluded from the analysis (kernel density hdPS curves for all patients included within the *Full Cohort* are provided in Additional file 2). Among the remaining 403,828 patients, we matched 116,014 patients (28.7 %) initiated on a high potency statin to 116,014 patients (28.7 %) initiated on a lower potency statin based on their individual hdPS; selected patients formed the *Matched hdPS Sub-Cohort* (Fig. 1).

Patients included within the *Matched hdPS Sub-Cohort* were on average 64.6 years old (SD 11.2) and 116,688 of them were males (50.3 %) (Table 3). Balance was obtained in all 18 examined patient characteristics, whether or not they were forced within the hdPS model (ASDD ranged from 0.001 to 0.023 with an average of 0.008).

**Table 3:** Demographic characteristics and comorbidity status of the *Matched hdPS Sub-Cohort* at baseline

	Low potency n (%)	High potency n (%)	Absolute standardized differences
	116,014 (100.0)	116,014 (100.0)	
Age, mean (SD)^a^	64.6 (11.2)	64.6 (11.2)	0.002
Male sex	58,194 (50.2)	58,494 (50.4)	0.005
At least 5 medical outpatient visits	66,453 (57.3)	66,390 (57.2)	0.001
At least 1 hospitalisation	28,265 (24.4)	28,604 (24.7)	0.007
Myocardial infarction	7558 (6.5)	7995 (6.9)	0.015
Stroke	3620 (3.1)	3897 (3.4)	0.013
Hypertension	48,268 (41.6)	48,474 (41.8)	0.004
Hypercholesterolemia	37,486 (32.3)	37,841 (32.6)	0.007
Peripheral vascular disease	2293 (2.0)	2671 (2.3)	0.023
Congestive heart failure	5198 (4.5)	5479 (4.7)	0.012
Coronary artery bypass graft	1670 (1.4)	1661 (1.4)	0.001
Percutaneous coronary intervention	4590 (4.0)	4846 (4.2)	0.011
Dispensation of loop diuretics	7139 (6.2)	7256 (6.3)	0.004
Dispensation of calcium blockers	26,510 (22.9)	26,716 (23.0)	0.004
Dispensation of beta-blockers	33,901 (29.2)	34,389 (29.6)	0.009
Dispensation of angiotensin receptor blockers	20,345 (17.5)	20,876 (18.0)	0.012
Dispensation of angiotensin converting enzyme inhibitors	24,472 (21.1)	25,289 (21.8)	0.017
At least 5 different drugs dispensed	66,600 (57.4)	66,820 (57.6)	0.004

Comorbidity status, drug dispensations and medical utilization rates were assessed in the year prior to the cohort entry date. Absolute standardized differences are defined as the between group difference over the pooled standard deviation of the two groups

^a^At the cohort entry date

### Performance of the PS and hdPS in adjusting for confounding by indication

**Fig. 2:**
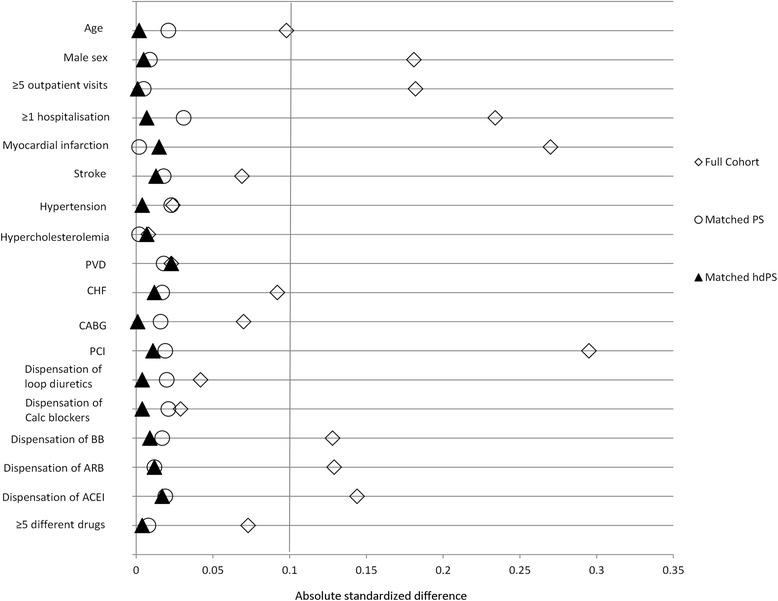
Comparison of the level of balance achieved using the absolute standardized differences obtained within the *Full Cohort*, the *Matched PS Sub-Cohort* and the *Matched hdPS Sub-Cohort* the examined patient characteristics. ACEI, Angiotensin converting enzyme inhibitors; ARB, Angiotensin receptor blockers; BB, Beta-blockers; CABG, Coronary artery bypass graft; Calc blockers, Calcium blockers; CHF, Congestive heart failure; hdPS, High-dimensional propensity score; PCI, Percutaneous coronary intervention; PS, Propensity score; PVD, Peripheral vascular disease

As mentioned previously, performance of both methods in adjusting for confounding by indication was tested by two distinct approaches, 1) by comparing the ASDD obtained within both sub-cohorts and 2) by comparing the adjusted OR of diabetes occurrence in the high over lower potency statin groups estimated by the logistic regression model used within the *Full Cohort* and both matched sub-cohorts. Figure 2 shows the direct comparison of the ASDD for the examined patient characteristics within the *Full Cohort*, the *Matched PS Sub-Cohort* and the *Matched hdPS Sub-Cohort*. Results indicate that both matched sub-cohorts were more balanced than the unmatched *Full Cohort*. Although the *Matched PS Sub-Cohort* provided greater balance on three of the 18 examined patient characteristics (MI, hypercholesterolemia, and PVD), overall, the *Matched hdPS Sub-Cohort* achieved the most balanced sub-cohort (average ASDD = 0.008 and average ASDD = 0.015 in the *Matched hdPS Sub-Cohort* and *Matched PS Sub-Cohort*, respectively).

Measures of associations obtained within the *Full Cohort* and the two matched sub-cohorts indicated that patients in the high potency group had higher odds of developing diabetes within 2-years follow-up. Results obtained within both sub-cohorts overlapped (OR = 1.10 [95 % CI 1.05–1.15] within the *Matched PS Sub-Cohort* and OR = 1.13 [95 % CI 1.08–1.19] within the *Matched hdPS Sub-Cohort*) but both were lower than those obtained within the *Full Cohort* (OR = 1.22 [95%CI 1.18–1.27]).

## Discussion

As expected, overall patient profiles within the *Full Cohort* showed imbalance on many key baseline characteristics suggesting the presence of confounding by indication. Such results would tend to indicate the presence of bias within measures of associations estimated within the *Full Cohort* if appropriate adjustment were not used in the analyses.

In their original paper, Schneeweiss *et al.* [7] assessed the performance of the hdPS method by comparing measures of associations adjusted for patients’ hdPS to the results of a RCT. By showing that the adjusted measures of association were closer to the results of the RCT than the crude measure of association, they showed that hdPS method had improved the adjustment for confounding by indication within their study. Performance of the hdPS method has been assessed by others using the same approach and their results also supported its use [12–14]. Measures of association obtained within both matched sub-cohorts were closer to the null (OR = 1.10 [95%CI 1.05–1.15] within the *Matched PS Sub-Cohort* and OR = 1.13 [95%CI 1.08–1.19] within the *Matched hdPS Sub-Cohort*) than within the *Full Cohort* (OR = 1.22 [95%CI 1.18–1.27]). However, since the CIs obtained from both methods overlap with each other and are as precise, their performance cannot be differentiated based solely on this criterion.

However, performance based on the level of balance achieved within matched sub-cohorts does not require an additional comparator. Based on this second performance criterion, we showed that using both methods created balanced matched sub-cohorts (i.e. ASDD were < 0.1 for all patient characteristics in both matched sub-cohort). When directly comparing both sub-cohorts, use of the hdPS method was favored since 14 out of the 18 examined patient characteristics were more balanced within the *Matched hdPS Sub-Cohort* than within the *Matched PS Sub-Cohort* which should tend to lead to less biased measures of association within the *Matched hdPS Sub-Cohort*. Seeing as the hdPS model adjusted for more variables than the PS model, such a result was to be expected but it needed to be verified in a situation where we have prior knowledge on which confounders to adjust for. The results we show in this study support the idea that the hdPS method may be used to adjust for confounding by indication, but the possibility that residual confounding remaining after this adjustment cannot be ruled out.

Our study has several strengths. *First*, we compared the PS and hdPS method in a large cohort of incident statin users showing substantial imbalance suggesting the potential for confounding by indication. As such, this provided an excellent situation in which to compare the performance of both methods.

*Second*, our conclusions favored the hdPS method when our study design should have favored the PS method. Although all of the examined covariates were forced within the PS model, only five investigator-selected covariates were forced within the hdPS model (only demographic, socio-economic and medical resource utilization variables were forced within the hdPS model, all remaining covariates were selected by the hdPS algorithm [n = 500]) [7]. Therefore, the hdPS method performance was mainly achieved through the use of the automated hdPS algorithm and not by investigator choice.

Our study has also several limitations. *First*, we compared patients on a relatively small number of baseline patient characteristics. It is possible that the performance observed within the 18 prespecified patient characteristics may not be representative of the overall performance regarding all potential patient characteristics. However, these variables were selected because we believed, like others have [18], that they could lead to confounding by indication and our results show that the hdPS method achieved substantial balance within all of these even though most were not forced within the hdPS model.

*Second*, we defined unbalance as ASDD > 0.1. Although this cut-off is frequently used [2, 10, 16], other values could have been used as well. Regardless of the cut-off value chosen, our results indicate that the hdPS method outperformed the PS method in achieving the most balanced sub-cohort. Although this added level of balance may not eliminate all biases within the observational study (i.e. information bias, unmeasured confounders, time-varying confounding), it will at least tend to reduce the level of bias caused by these baseline characteristics.

*Third*, no mechanism of action by which statins could cause diabetes has been identified. Although we compared both methods using a frequently used exposure definition, we cannot claim that this exposure definition reflects the true mechanism of action by which statins could cause diabetes. It is possible that the results obtained, had we used an exposure definition reflecting the true mechanism of action, could have differed from those obtained within this study. This also reflects the fact that we do not know what the true measure of association between the exposure to statins and diabetes is. As mentioned, traditionally the hdPS method has been validated by comparing the crude and hdPS-adjusted measures of association to a gold standard measure but in this case, such a true gold standard is not available. We recognize that this would not have been the case had we conducted this comparison within an ordinary simulation study in which the truth would be defined by the investigators. However, as others have highlighted, [7, 30] the hdPS method cannot be evaluated through the use of ordinary simulation studies since its performance depends on the complexity and quantity of data available to the hdPS algorithm, levels of which cannot be reproduced within a fully artificial setting. In order to circumvent this issue, Franklin et al. [30] recently proposed that performance of the hdPS method compared to the performance of the PS method be compared using plasmode simulation studies. Using this framework, Franklin et al. showed that an investigator-independent hdPS method performed nearly as well as a fully specified PS model further supporting the use of the hdPS method in situations where little prior knowledge regarding potential confounding variables is available [30]. Such an approach may be validated in future work aimed at further evaluating the performance of the hdPS method.

*Fourth,* our results show that hdPS trimming removed slightly more patients than PS trimming (301 vs 55). Although this could impact our conclusion regarding the value of both methods, its impact should be marginal since the total number of patients that were trimmed in both settings remains trivial in comparison to the total sample size of the *Full Cohort*.

*Finally*, we only examined the relative performance of the PS and hdPS methods within a single context; the results obtained within this study may not be generalizable to other studies focusing on other exposure-disease associations. Furthermore, we only compared the traditional PS method estimated using a logistic regression model to hdPS method, while other methods are also available (e.g., classification and regression trees and boosting methods) [31, 32]. Future work will be needed to compare the relative performance of all these different methods.

## Conclusions

In conclusion, we recommend comparing the PS and hdPS methods by means of their relative ability to select balanced sub-cohorts over their adjustment potential within ethiological studies. Although both methods adequately adjusted for confounding by indication, we cannot rule out the possibility that the hdPS method will be dominant in other contexts since it has the potential to identify confounders which are unknown to the investigators.

### Ethics approval and consent to participate

Access to data was granted by the *Commission d’accès à l’information* and the protocol was approved by the *Centre hospitalier de l’Université de Montréal* ethics’ committee.

### Consent for publication

Not applicable

## Additional files

Additional file 1:
**Kernel density curves of the PS distribution within the** ***Full Cohort.*** (TIF 261 kb) Additional file 2:
**Kernel density curves of the hdPS distribution within the** ***Full Cohort.*** (TIF 460 kb)

## Abbreviations

ASDD: absolute standardized differences
CI: confidence interval
hdPS: high-dimensional propensity score
MED-ECHO: Maintenance et Exploitation des Données pour l’Étude de la Clientèle Hospitalière
MI: myocardial infarction
OR: odds ratio
PCI: percutaneous coronary intervention
PS: propensity score
PVD: peripheral vascular disease
RAMQ: Régie de l’assurance maladie du Québec
RCT: randomized controlled trials
SD: standard deviation

## References

[1] Groenwold RH, Hak E, Hoes AW (2009). Quantitative assessment of unobserved confounding is mandatory in nonrandomized intervention studies. J Clin Epidemiol.

[2] Austin PC (2011). An introduction to propensity score methods for reducing the effects of confounding in observational studies. Multivar Behav Res.

[3] Shrank WH, Patrick AR, Brookhart MA (2011). Healthy user and related biases in observational studies of preventive interventions: a primer for physicians. J Gen Intern Med.

[4] Austin PC (2009). The relative ability of different propensity score methods to balance measured covariates between treated and untreated subjects in observational studies. Med Decis Making.

[5] Rosenbaum PR, Rubin DB (1983). The central role of the propensity score in observational studies for causal effects. Biometrika.

[6] Black CM, Tadrous M, Cadarette SM. Diffusion of methodological innovation in pharmacoepidemiology: high-dimensional propensity score co-authorship network analysis. CAPT; Toronto: J Popul Ther Clin Pharmacol. 2013;21(1):e138.

[7] Schneeweiss S, Rassen JA, Glynn RJ, Avorn J, Mogun H, Brookhart MA (2009). High-dimensional propensity score adjustment in studies of treatment effects using health care claims data. Epidemiology.

[8] Belitser SV, Martens EP, Pestman WR, Groenwold RHH, de Boer A, Klungel OH (2011). Measuring balance and model selection in propensity score methods. Pharmacoepidemiol Drug Saf.

[9] Austin PC (2009). Balance diagnostics for comparing the distribution of baseline covariates between treatment groups in propensity-score matched samples. Stat Med.

[10] Mamdani M, Sykora K, Li P, Normand SL, Streiner DL, Austin PC (2005). Reader’s guide to critical appraisal of cohort studies: 2. Assessing potential for confounding. BMJ.

[11] Ali MS, Groenwold RHH, Pestman WR, Belitser SV, Roes KCB, Hoes AW (2014). Propensity score balance measures in pharmacoepidemiology: a simulation study. Pharmacoepidemiol Drug Saf.

[12] Garbe E, Kloss S, Suling M, Pigeot I, Schneeweiss S (2012). High-dimensional versus conventional propensity scores in a comparative effectiveness study of coxibs and reduced upper gastrointestinal complications. Eur J Clin Pharmacol.

[13] Polinski JM, Schneeweiss S, Glynn RJ, Lii J, Rassen JA (2012). Confronting “confounding by health system use” in Medicare Part D: comparative effectiveness of propensity score approaches to confounding adjustment. Pharmacoepidemiol Drug Saf.

[14] Rassen JA, Glynn RJ, Brookhart MA, Schneeweiss S (2011). Covariate selection in high-dimensional propensity score analyses of treatment effects in small samples. Am J Epidemiol.

[15] Preiss D, Seshasai SR, Welsh P, Murphy SA, Ho JE, Waters DD (2011). Risk of incident diabetes with intensive-dose compared with moderate-dose statin therapy: a meta-analysis. JAMA.

[16] Ko DT, Wijeysundera HC, Jackevicius CA, Yousef A, Wang J, Tu JV (2013). Diabetes and cardiovascular events in older myocardial infarction patients prescribed intensive-dose and moderate-dose statins. Circ Cardiovasc Qual Outcomes.

[17] Carter AA, Gomes T, Camacho X, Juurlink DN, Shah BR, Mamdani MM (2013). Risk of incident diabetes among patients treated with statins: population based study. BMJ.

[18] Dormuth CR, Filion KB, Paterson JM, James MT, Teare GF, Raymond CB (2014). Higher potency statins and the risk of new diabetes: multicentre, observational study of administrative databases. BMJ.

[19] Wang K-L, Liu C-J, Chao T-F, Huang C-M, Wu C-H, Chen S-J (2012). Statins, risk of diabetes, and implications on outcomes in the general population. J Am Coll Cardiol.

[20] Zaharan NL, Williams D, Bennett K (2013). Statins and risk of treated incident diabetes in a primary care population. Br J Clin Pharmacol.

[21] Soucy A. Québec Handy Numbers, 2015 Edition. Québec: Institut de la statistique du Québec; 2015.

[22] Blais C, Lambert L, Hamel D, Brown K, Rinfret S, Cartier R (2012). Évaluation des soins et surveillance des maladies cardiovasculaires: Pouvons-nous faire confiance aux données médico-administratives hospitalières ?.

[23] Lambert L, Blais C, Hamel D, Brown K, Rinfret S, Cartier R (2012). Evaluation of care and surveillance of cardiovascular disease: can we trust medico-administrative hospital data?. Can J Cardiol.

[24] Tamblyn R, Lavoie G, Petrella L, Monette J (1995). The use of prescription claims databases in pharmacoepidemiological research: the accuracy and comprehensiveness of the prescription claims database in Quebec. J Clin Epidemiol.

[25] Tamblyn R, Reid T, Mayo N, McLeod P, Churchill-Smith M (2000). Using medical services claims to assess injuries in the elderly: sensitivity of diagnostic and procedure codes for injury ascertainment. J Clin Epidemiol.

[26] World Health Organisation Collaborating Centre for Drug Statistics Methodology. ATC/DDD Index [July 23rd 2014]. Available from: http://www.whocc.no/atc_ddd_index/.

[27] Law MR, Wald NJ, Rudnicka AR (2003). Quantifying effect of statins on low density lipoprotein cholesterol, ischaemic heart disease, and stroke: systematic review and meta-analysis. BMJ.

[28] Sturmer T, Rothman KJ, Avorn J, Glynn RJ (2010). Treatment effects in the presence of unmeasured confounding: dealing with observations in the tails of the propensity score distribution--a simulation study. Am J Epidemiol.

[29] Austin PC (2011). Optimal caliper widths for propensity-score matching when estimating differences in means and differences in proportions in observational studies. Pharm Stat.

[30] Franklin JM, Schneeweiss S, Polinski JM, Rassen JA (2014). Plasmode simulation for the evaluation of pharmacoepidemiologic methods in complex healthcare databases. Comput Stat Data Anal.

[31] Westreich D, Lessler J, Funk MJ (2010). Propensity score estimation: neural networks, support vector machines, decision trees (CART), and meta-classifiers as alternatives to logistic regression. J Clin Epidemiol.

[32] Lee BK, Lessler J, Stuart EA (2010). Improving propensity score weighting using machine learning. Stat Med.

