# Supplementary figures and images for: Head to head comparison of the propensity score and the high-dimensional propensity score matching methods
Source: BMC Med Res Methodol. 2016 Feb 19;16:22. doi: 10.1186/s12874-016-0119-1 (PMC4759710; doi:10.1186/s12874-016-0119-1)

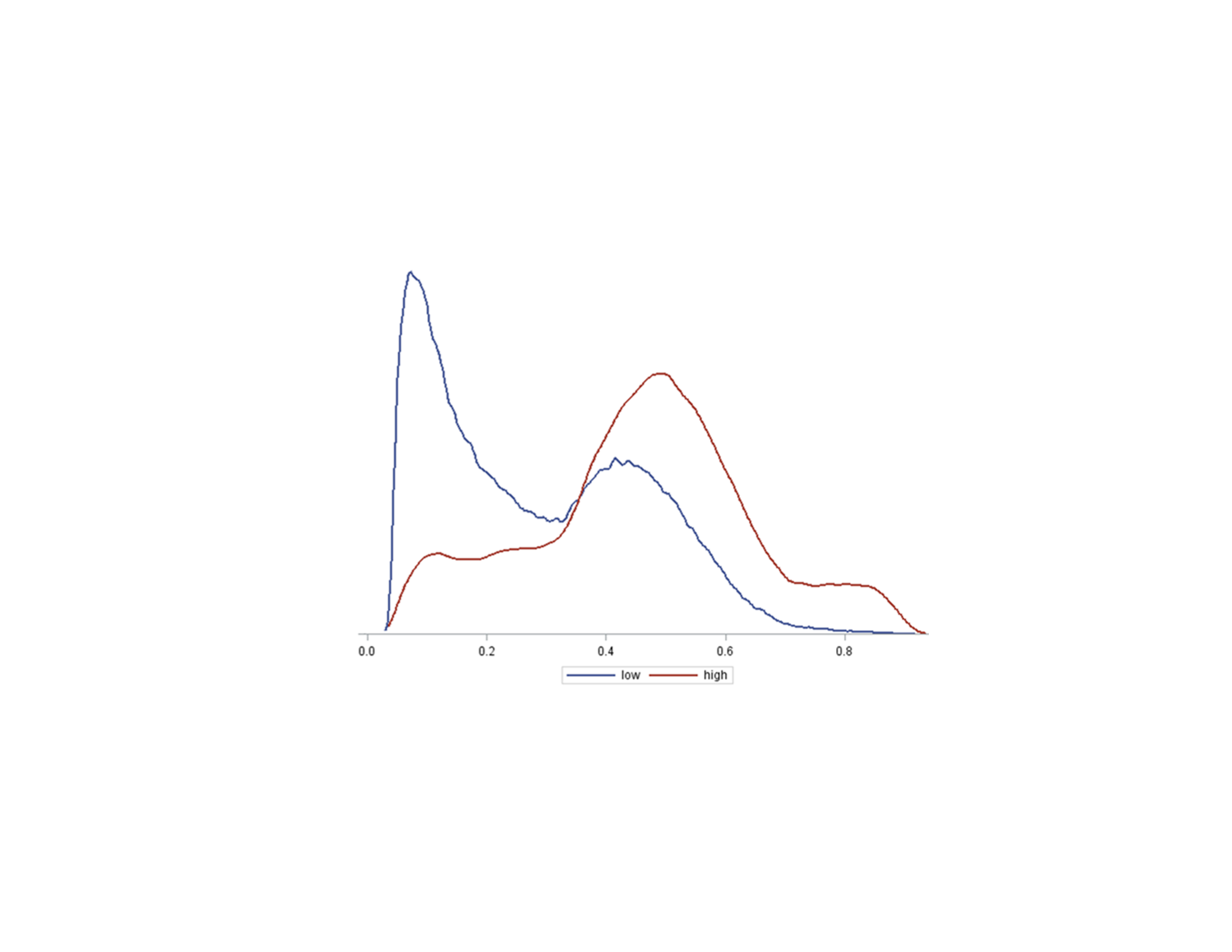

Supplement: Additional file 1: — Kernel density curves of the PS distribution within the Full Cohort. (TIF 261 kb) [file 12874_2016_119_MOESM1_ESM.tif]

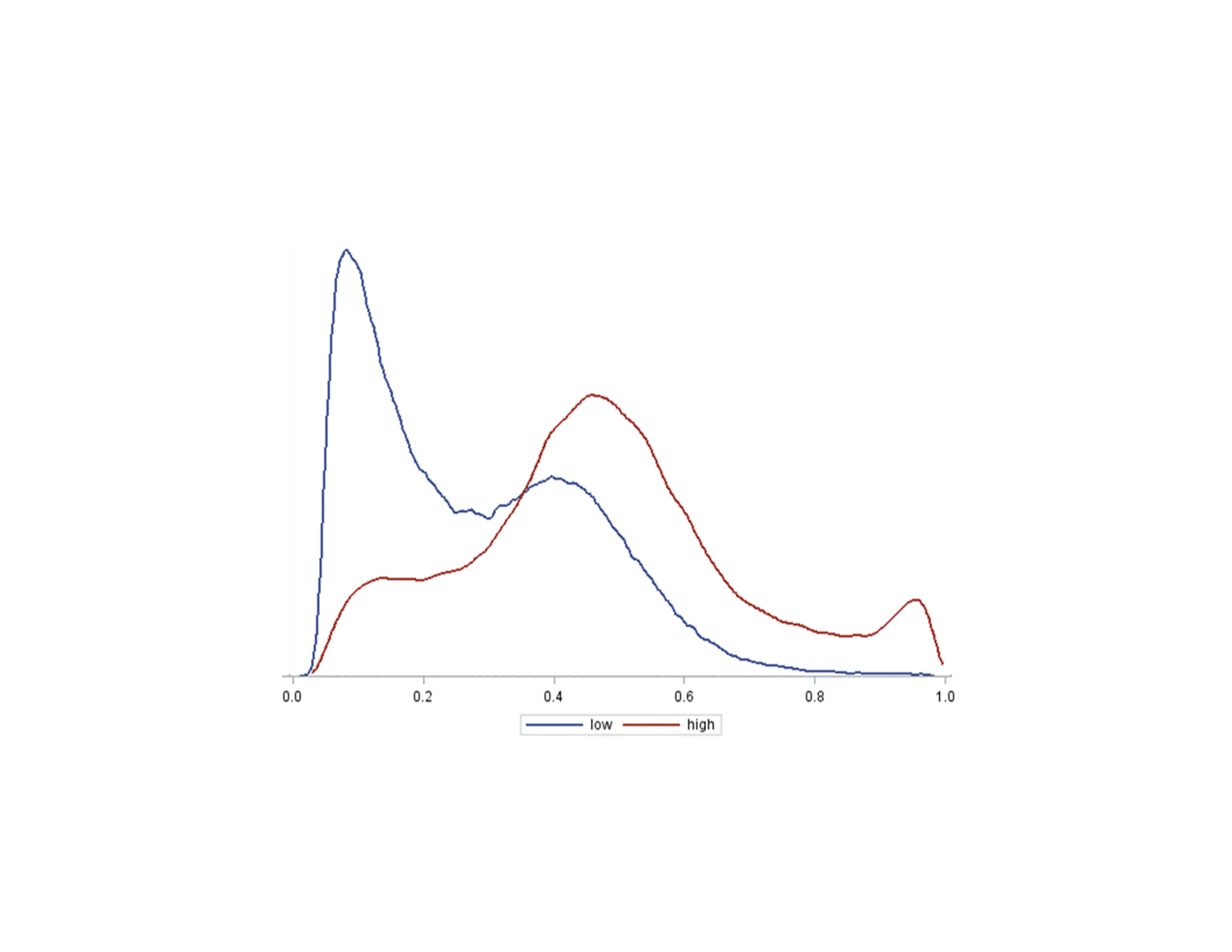

Supplement: Additional file 2: — Kernel density curves of the hdPS distribution within the Full Cohort. (TIF 460 kb) [file 12874_2016_119_MOESM2_ESM.tif]
